# Blood donor safety, prevalence and associated factors for cytomegalovirus infection among blood donors in Minna-Nigeria, 2014

**DOI:** 10.11604/pamj.supp.2019.32.1.13297

**Published:** 2019-01-22

**Authors:** Musa Kalamullah Bawa, Aisha Mamman, Adebola Olayinka, Saheed Gidado, Ndadilnasiya Endie Waziri, Muhammad Shakir Balogun, Kabir Ibrahim Getso, Mahmood Muazu Dalhat, Peter Nsubuga, Nuruddeen Aliyu, Hussaini Bala, Hauwa Muhammad, Suleiman Haladu, Usman Lawan Shehu, Patrick Mboya Nguku

**Affiliations:** 1 Nigeria Field Epidemiology and Laboratory Training Program, Abuja-Nigeria; 2 Ahmadu Bello University, Zaria, Nigeria; 3 Ministry of Health, Kano, Nigeria; 4 Global Public Health Solutions, Atlanta GA, USA

**Keywords:** Cytomegalovirus, blood donors, Minna, Northern Nigeria

## Abstract

**Introduction:**

human cytomegalovirus (CMV) has remained a cause of morbidity and mortality in pregnancy and immunocompromised patients. CMV is transmissible through blood transfusion. We conducted a descriptive, cross-sectional study to assess blood donor safety and to determine the prevalence and associated factors for CMV infection among blood donors in Minna, Nigeria.

**Methods:**

all consenting blood donors were screened for CMV antibodies (IgM and IgG) using ELISA kit and haematological indices using a haematological analyzer. We administered structured questionnaires to obtain socio-demographic and socio-economic data. Data were subjected to univariate, bivariate and multivariate statistical analyses using Epi Info version 3.5.4. Significant associations were presumed if p < 0.05.

**Results:**

a total of 345 participantswere recruited, the majority were males 336 (97.4%). Monthly earnings of majority of the blood donors, 136 (40.6%) ranged from ₦18,000 to ₦35,000. The prevalence of CMV infection was 96.2%. The prevalence of anti-CMV IgG antibodies was 96.2% and that of IgM was 2.6%. Most of the study participants, 274 (79.4%) were family replacement donors. The majority of the blood donors 195 (56.5%) were anaemic (PCV < 36, Hb < 12g/dl). Those with positive CMV were more likely to be of high-income level (OR = 0.32, P = 0.04).

**Conclusion:**

the seroprevalence of CMV was high with a significant proportion of donors capable of transmitting CMV infection to blood recipients. The majority of the blood donors were anaemic. High income level is associated with CMV infection. Quality of screening for anemia be improved.

## Introduction

Blood transfusion is usually a lifesaving therapeutic intervention. However, many preventable errors may make this a hazardous procedure [1]. The World Health Organization (WHO) recommends that blood donation should in all cases be voluntary [2]. However, in Nigeria, voluntary donors are relatively scarce. Hence, family replacement and commercial donors have become alternative sources of blood [3]. Healthy persons who are between the ages of 18 and 65 years with haemoglobin (Hb) levels of not less than 13.5 g/dl in males or 12.5 g/dl in females are acceptable as donors if they test negative for transfusion-transmissible infections (TTIs). These TTIs include hepatitis B virus (HBV), hepatitis C virus (HCV), human immunodeficiency virus (HIV), malaria, syphilis and chagas disease. However, females are only accepted as donors if they are not pregnant or breastfeeding [4]. Human cytomegalovirus (CMV), otherwise called human herpes virus type 5, over the years, has come to assume an important public health problem. As it is a significant cause of morbidity and mortality in pregnancy and among immunocompromised patients like recipients of organ transplants, HIV-infected persons, cancer patients on therapy and neonates [5].

Cytomegalovirus is transmissible through blood transfusion, among other parenteral routes [6]. However, donor screening for CMV is not routinely undertaken in Nigeria. A study by Chakravarti shows that most adults across the globe are seropositive for CMV [6]. The African region of the WHO, fraught with high disease burden and high prevalence of TTIs is faced with unique challenges to blood safety [7]. CMV is part of this challenge. A study in Ghana reported a CMV prevalence of 93.2% [7]. A previous study on the pre-donation screening of intending blood donors for antibodies to infectious agents at Obafemi Awolowo University Teaching Hospital Complex, Ile-Ife, Osun State, Nigeria indicated that about 12% donors were deferred due to TTIs and 5% due to anaemia [8]. The prevalence of transmissible transfusion viruses is still very high in Nigeria when compared with other developing countries with very similar challenges [9]. This challenge needs to be addressed to ensure blood and blood product safety. CMV prevalence rates in some parts of Nigeria were from 92% in Jos to 95% in Lagos both in 2009 [10].

The symptoms of CMV infection vary depending on the age and health of the person who is infected, and how the infection occurred and include hearing, vision, neurological and developmental problems. Other symptoms and morbidities of CMV include premature delivery, jaundice, spleen, microcephaly (small head), seizures, rash and feeding difficulties [8]. Premature and ill full-term infants who are infected soon after birth are also at risk for neurological and developmental problems over time [8, 10]. Transfusion transmitted-cytomegalovirus (TT-CMV) is a significant cause of morbidity and mortality in the immunocompromised host. The risk of TT-CMV from seropositive donors is reported to be 0.4 to 12% [11]. The blood transfusion screening algorithm in Minna, Niger State includes HIV, HBV, HBC and syphilis. Malaria is endemic and anaemia is a common diagnosis in the area [12]. We conducted a study to determine the prevalence of and associated risk factors for CMV infection in blood donors in Minna, Northern Nigeria.

## Methods

**Study area:** the study was conducted in Minna, the capital city of Niger State, Nigeria. The city is located in the North Central region of Nigeria. Minna City is made up of two local government areas (LGAs), each having one secondary health facility, General Hospital (GH), Minna and Ibrahim Babangida Specialist Hospital (IBBSH), Minna located in Minna South and Minna East LGAs respectively. The average annual blood donor rates were 3,865 blood donors for GH, Minna and 756 for IBBSH, Minna. The population of Minna is 304, 113 projected from 2007 census.

**Study design:** we conducted a hospital-based cross-sectional study with both descriptive and analytical components. The descriptive component described the occurrence of CMV among the blood donors regarding person, place and time, while the analytical component identified the factors associated with CMV infection**.**

**Study population:** the study population was all potential blood donors that presented at the identified blood donation centers, GH Minna and IBBSH, Minna, Niger State.

**Eligibility criteria:** we included apparently healthy looking persons who were between the ages of 18 and 65 years with haemoglobin (Hb) levels of not less than 13.5 g/dl in males or 12.5 g/dl in females and those already screened (laboratory screening) for transmission transmissible infections and found eligible to donate. The TTIs considered excluded CMV as donor screening for CMV is not routinely undertaken in Nigeria. Apparently healthy looking persons were considered to be people apparently looking well fed devoid of physically observable signs of sickness or disease.

**Exclusion criteria:** all those that tested positive for HIV, HBV and HCV were excluded.

**Sample size determination:** a total of 345 blood donors were recruited into the study. The sample size was obtained using the following formula:

$$n=\frac{z\propto^{2}pq}{d^{2}}$$

**Where:** n= required sample size, our Z (z = ^(1-a/2)^) value was 1.96. This represents the value of the standard distribution corresponding to a significance level of α (1.96 for a 2-sided test at the 0.05 level). We used a prevalence (p) of 92% (0.92) obtained from CMV prevalence study among prospective blood donors at a tertiary health facility (Jos University Teaching Hospital) in Jos, Nigeria [5]. Our q (1-p) was 0.08 and our absolute precision (d) was 3% (0.03).

$$\text{n}=\frac{1.96^{2}\times 0.92\times 0.08}{0.03^{2}}=314$$

Using 10% non-response obtained as 31, we obtained the sample size (n) 345.

### Sampling technique

We employed systematic random sampling technique. We proportionately allocated the participants to the health facilities, GH, Minna and IBBSH, Minna using the sampling frames for the health facilities. The blood donors that fitted into the inclusion criteria of the study and consented to the study were systematically selected from the two health facilities after obtaining an interval for selection for each, using n/N = K. The interval was 5 and the first participant was selected randomly by balloting between 1 and 5, and then after that every Kth blood donor recruited until the required sample was obtained.

### Study instruments

We used laboratory forms for capturing laboratory data, and interviewer-administered questionnaires made up of demography and risk factor sections were used for capturing data on socio-demographic, socio-economic characteristics and practices of the participants.

### Data collection methods

Data were collected by trained research assistants and laboratory scientists. The data collection was from November 2013 to January 2014. 5ml of whole blood was collected using EDTA and serum using plain vacutainers from each subject. The serum was used for CMV ELISA screening for CMV antibodies, i.e. IgG and IgM using ELISA kit (DIALAB® Austria), while the whole blood was analyzed for haematological indices using a haematological analyzer (*Abacus junior* haematology *analyzer 2.75*, manufactured in 1995 by Diatron® U.S.A). We used interviewer-administered questionnaires to obtain information from participants regarding their socio-demographic, socio-economic characteristics and practices [8].

### Screening for CMV

Screening for CMV antibodies was done using ELISA kit (DIALAB® Austria). Serum was preserved under -20°C and analyzed using ELISA. The analysis was done using automated microplate reader (*Emax*precision microplate reader), E11865 model (*Molecular Devices®* USA). ELISA procedure is shown in detail in an attached Annex 1. No molecular testing was done.

### Analysis for haematological indices

Blood samples for haematological analyses were analyzed daily within 12 hours of collection using a haematological analyzer (*Abacus junior* haematology *analyzer 2.75*). The procedure included: 1) Using the EDTA blood container, whole blood was inserted into the analyzer; 2) The analyzer aspirated 2 micro liters of the blood into the probe and autoanalysed; 3) The result appeared on the screen and copied.

### Data management

The independent variables were the socio-demographic and socio-economic characteristics of the respondents, cultural practices, while the dependent variable was the CMV serological status of the blood donors regarding IgM and IgG, which are either positive or negative. The socio-demographic and socio-economic characteristics included age, sex, occupation, educational status, ethnic group, marital status, past history of transfusion, type of donor and monthly income level. The cultural practices included tribal marks, received blood transfusion, received surgical procedure in HF, local circumcision, local *belubelu* (uvulectomy), dental procedure inside HF, dental procedure outside HF, worked in contact with blood and sex partner not spouse.

We reviewed all the completed questionnaires before electronic entry. Data obtained were analyzed using Epi Info version 3.5.4 (US Centers for Disease Control and Prevention) and Microsoft Excel 2007. Significant associations were presumed if p < 0.05.

### Ethical considerations

Ethical clearance was obtained from the GH, Minna and IBBSH, Minna researchand ethical clearance committees. Respect to participants’ rights was observed including the right to refuse participation with explanation through participant’s information form. We conducted informed consent for all potential participants prior to study through provision of individual consent forms for their consent.

## Results

**Figure 1:**
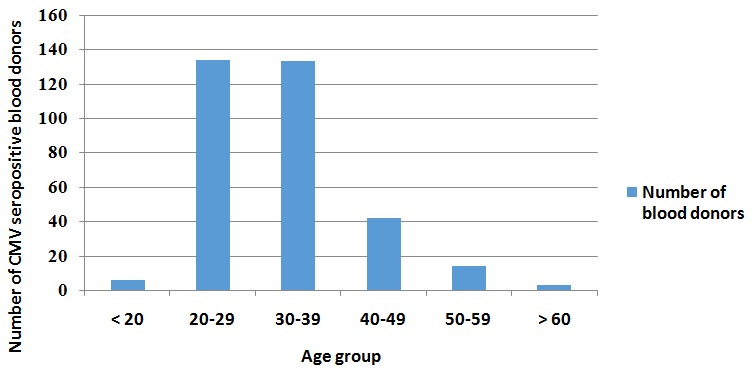
showing the frequency of CMV seropositivity by age group of the blood donors in Minna, Nigeria

The majority of the blood donors, 139 (40.3%) were aged 20-29 years, 230 (66.7%) were married, 65 (19.5 %) were unemployed, 273 (79.4%) were family blood donors. Most, 146 (42.6%) had post-secondary education, and 136 (40.6%) had a monthly income of between $51 and $100 (Table 1). The prevalence of CMV infection was found to be 96.2%. The prevalence of CMV IgG was found to be 96.2%, and that of CMV IgM was 2.6%. Combined CMV IgG and CMV IgM antibodies were detected in 9 (2.6%) blood donors (Table 2). Analyses of the distribution of CMV IgG seropositivity with age showed blood donors aged 20-29 years had the highest CMV IgG seroprevalence, 40.4% closely followed by those aged 30-39 years with 40.1%. The least CMV IgG seroprevalence, 0.9% was found in the blood donors aged > 60 years (Figure 1).

**Table 1:** sociodemographic characteristics of blood donors in Minna-Nigeria, 2014

Variable	Frequency (N=345)	Percent (%)
**Age**		
< 20	6	1.7
20 - 29	139	40.3
30 - 39	137	39.7
40 - 49	46	13.3
50 - 59	14	4.1
≥ 60	3	0.9
**Marital status**		
Married	230	66.7
Single	113	32.8
Separated	2	0.6
**Occupation**		
Self employed	183	53.0
Employed	95	27.5
Student	59	17.1
Unemployed	6	1.7
Retired	2	0.6
**Educational status**		
Postgraduate	38	11.1
Tertiary (first degree)	108	31.5
Secondary	96	28.0
Primary	26	7.6
No formal education	75	21.9
**Monthly income level**		
Above N120000	3	0.9
N70000 - N20000	16	4.8
N35000 - Below N70000	91	27.2
N18000 - Below N35000	136	40.6
Below N18000	89	26.6
**Type of donor**		
Commercial	11	3.2
Voluntary	60	17.4
Family	273	79.4

**Table 2:** seroprevalence of anti-CMV IgG and IgM antibodies among blood donors in Minna-Nigeria, 2014

	Anti-CMV IgG		Anti-CMV IgM	
Sero-status	Frequency (N-345)	Percent (%)	Frequency (N=345)	Percent (%)
Positive	332	96.2	9	2.6
Negative	13	3.8	336	97.4
Total	345	100	345	100

Bivariate analysis for socio-economic factors for CMV (IgG and IgM) infection showed blood donors with monthly income level < ₦18000 were less likely to be CMV-positive than those with higher income (OR = 0.32 (95% confidence interval: 0.10-0.97)). Other factors, age, sex, marital status, type of marriage, educational level, and occupation showed various levels of association but were not significant (p > 0.05) (Table 3). Table 4 shows the pattern of haemoglobin concentration (Hb) and packed cell volume (PCV) among various strata of blood donors. Of the 345 blood donors, 227 (65.8%) had Hb below the normal range (i.e. Hb < 12g/dl). Family replacement blood donors formed the majority, 185 (81.5%) of the anaemic blood donors. Most blood donors (197, 56.5%) had PCV below the normal range (i.e. PCV less than 36%). None of the blood donors had Hb or PCV above the normal range. Analysis for variations and statistical significance of means of CMV seropositive group and CMV seronegative group on haematological indices showed CMV seropositive group had a low Hb (p = 0.02), PCV (p = 0.03) and mean platelet distribution width (MPDW) (p = 0.04) compared to those of CMV seronegative group (Table 5).

**Table 3:** socioeconomic-associated factors for CMV (IgG) infection among blooddonors in Minna-Nigeria, 2014

Variable	CMVI Gg			
	Positive	Negative	OR (95%CI)	P-value
**Marital Status**				
Married	224 (67.5)	6 (46.2)	2.42 (0.79-7.37)	0.09+
Single	108 (32.5)	7 (53.8)		
**Type of marriage**				
Polygamous	56	57	1.76 (0.20-15.4)	0.51+
Monogamous	169	164		
**Sex**				
Male	323 (97.3)	13 (100)	1.26 (0.07-22.81	0.68+
Female	9 (2.7)	0 (0.0)		
**Age**				
< 40 years	288 (86.7)	11 (84.6)	1.19 (0.26-5.55)	0.54+
≥ 40 years	44 (13.3)	2 (15.4)		
**Monthly income level**				
<N18000	90 (27.1)	7(53.8)	0.32 (0.10-0.97)	0.04+
≥N18000	242 (72.9)	6 (46.2)		
**Educational level**				
Low education	98 (29.6)	3 (23.1)	1.40 (0.38-5.20)	0.44+
High education	233 (70.4)	10 (76.9)		
**Occupation**				
Unemployed	63 (19.0)	4 (30.8)	0.53 (0.16-1.77)	0.23+
Employed	269 (81)	9 (69.2)		

+ Fisher’s exact test

**Table 4:** pattern of Hb and PCV among various strata of blood donors in Minna-Nigeria, 2014

Category	Type of blood donor	Parameters			
		Hb		PCV	
		Frequency	Percentage	Frequency	Percentage
**Low**	Commercial	8	3.5	6	3.1
	Family	185	81.5	158	81.0
	Voluntary	34	15.0	31	15.9
**Normal**	Commercial	3	2.5	5	3.3
	Family	89	75.4	116	77.4
	Voluntary	26	22.0	29	22.0

**NB-Values of normal ranges** Hb: 12-17.4 g/dl, PCV: 36-52%. Hb-Low: <12g/dl, Normal: 12-17.4g/dl, High: >17.g/dl. PCV- Low: <36%, Normal: 36-52%, High: >52%

**Table 5:** variations and statistical significance of means of CMV seropositive and CMV seronegative groups of blood donors in Minna, North Central Nigeria

Variable	Means and standard deviation				*P-value*
	CMV seropositive group		CMV seronegative group		
	Means	Standard deviation	Means	Standard deviation	
Hb	11.31	0.08	11.38	1.67	0.02
Lymphocyte	33.72	13.17	31.41	12.80	0.59
WBC	7.10	2.56	6.81	2.34	0.72
PCV	34.85	5.32	34.88	2.74	0.03
Platelet	286.44	107.28	251.36	89.08	0.25
RBC	4.63	0.48	4.36	0.74	0.27
MCH	25.61	1.90	25.73	2.99	0.91
Neutrophil	57.18	13.99	57.50	14.56	0.95
MIE	8.7	3.58	10.86	5.56	0.25
MPV	8.81	2.25	8.06	1.37	0.10
MCV	81.22	6.36	79.26	7.26	0.42
MCHC	32.23	0.97	32.45	1.42	0.64
MCDW	15.76	1.58	15.23	2.19	0.47
MPDW	32.37	8.30	36.02	5.229	0.04

## Discussion

We aimed at assessing the blood donor safety and determining the prevalence and associated factors for CMV infection among blood donors in Minna, Nigeria.We found the seroprevalence of CMV to be 96.2%; this is consistent with findings of a study in Sfax region, Tunisia (97.1%), Jos, Nigeria (92%), and Lagos, Nigeria (96%) [5, 11, 12]. The finding indicates CMV infection is widely spread among the human population but not commonly known as most CMV infections are asymptomatic and therefore commonly go undiagnosed [13]. The high prevalence of anti-CMV IgG antibodies found in this study corroborates findings of other studies in Southern Brazil (96.4%),Ghana (93.2%), Ilorin (96.7%), and Lagos, Nigeria (96%) [8, 12-14]. We found the prevalence of anti-CMV IgM antibodies as 2.6%; this is lower than 5.5% reported at Albania and 3.1% at Benin, Nigeria [10]. Studies with nearly comparable anti-CMV IgM antibody rates include 2.3% in Brazil and 2.5% in Sudan [15, 16]. CMV IgM prevalence represents the proportion of those with reactive or ongoing CMV infection and are capable of infecting others: This reflects the role of CMV as a TTI [14].

Combined CMV IgG and CMV IgM antibodies were detected in 9 (2.6%) participants, a figure that is higher than 1.6% reported by Chaudhari and Bindra [17]. Re-activation infection is associated with a CMV IgM response. Individuals who suffer a re-activation have previously mounted a CMV IgG response. Therefore, anti-CMV IgG prevalence rates are considered to reflect the overall prevalence for epidemiologic purposes; as CMV IgG reflects a chronic infection [5, 6, 14]. CMV IgG seropositivity distribution with age showed CMV seroprevalence deferred with age. We found the blood donors aged 20-29 years had the highest CMV seroprevalence of 40.4% closely followed by those aged 30-39 years, 40.1% while the least was found in those aged > 60 years as 0.9%. Conversely, younger blood donors aged < 20 had CMV seroprevalence of 1.8%. This is very similar with the finding by Alao where the peak age CMV seroprevalence was in blood donors aged 25-29 years age, which represented 30.4% of the study participants. Our finding is also closely similar with another study which had the highest CMV serprevalence in blood donors aged 30-39 as we found the age group 30-39 years ranked the second highest CMV seoprevalence [10]. CMV seroprevalence was lowest in those aged 15-19 years and above 50 years(1.6% each) [5]. However, our finding contrast a finding by Wujcicka and others which indicates that CMV seropositivity is significantly associated with increasing age [18]. Given that CMV typically leads to lifelong seroconversion, it will be expected that CMV seroprevalence will increase with age. The difference in the findings might have to do with the study populations and need to be explored further.While our study population was potential blood donors that presented at the identified blood donation centers aged between 18 to 65 years, the study by Wujcicka and others was in a cohort of pregnant Polish women.

Our study found family replacement donors constituted the majority, 79.4% while commercial donors made up 3.2% and voluntary donors 17.4%. The low proportion of voluntary donors reflects the duo role of ignorance and low national development index [2]. Family replacement blood donation predominates in the absence of a well organized national voluntary blood donation programme. People then rely on family or friends of patients to act as replacement donors. However, research findings indicates that blood from family or replacement donor is found to be unsuitable more often than blood from voluntary non enumerated and therefore presents a potentially greater risk to the safety of the blood supply [19, 20]. The commonest age group in this study was 20-29 years; this is higher than 18-20 years reported by a study in Chennai, India [21]. Our finding showed an overwhelming male predominance (97.4%). It is comparable to 95.4% reported by Akinbami and colleagues [14]. It maybe linked to the belief that women do not donate blood because of menstrual flow, pregnancy and childbearing [22].

We found that most study participants were employed, retirees accounted for very few. It shows blood donation is an activity of persons < 65 years of age [2]. The majority of the blood donors were educated as those without education were 21.9%. The rate of illiteracy observed in our study is higher than 15.4% reported for the North-Central zone in the National Demographic and Health Survey (NDHS) 2008 by National Population Commission, Nigeria (NPC) [23]. People earning of ₦18,000 and above were more likely to be CMV antibody positive. Blood donors with a monthly income level less than ₦18,000 were 68% less likely to be CMV-positive than those with monthly income level equal to or higher than ₦18,000. Our finding is consistent with findings by Revello and Giuseppewhere they found that the risk of primary maternal infection of CMV was about three times higher among the higher-income susceptible women (45%), compared to 15% in the lower-income group [24]. This finding is contrary to the report in California in which persons who earned less than $1,000 had a risk of 43.5% more than individuals with higher income [25]. Our finding also contrasts other findings that showed that the major risk factor for CMV infection is exposure to children [18, 26-28]. Many of the blood donors in this study, 55.9% were found to be anaemic (PCV < 36%). The study participants were considered to have been screened (laboratory screening) for anaemia with PCV cutoffs as not less than 40.5% (13.5 g/dl) in males or 37.5% (12.5 g/dl) in females based on the blood donor eligibility criteria of the country. Yet, such a high level of proportion of anaemic blood donors was found. This goes to show the level of the quality issues associated with screening procedures. The high proportion of anaemia in our study corroborates findings of other studies which include studies in Port Harcourt, Nigeria and south India [29, 30]. This is also identical with findings of Xu and colleagues, Gordon-smith and associates and Taglietti and colleagues [31-33]. This high level of anaemia may be associated with the finding in the study that 81.5% of the anaemic donors were family replacement donors and family replacement donors constituted 79.4% of all the donors (345) in the study.

Comparison of the difference of the means of PCV and Hb between CMV seropositive and CMV seronegative donors showed CMV seropositive donors had a lower PCV and Hb (P < 0.05). CMV seropositivity with its attendant risk predisposes to anaemia [34]. CMV causes infection of bone marrow suppression which is a risk factor for aplastic anaemia [35-37]. The findings of our study cannot be generalized to Nigeria as the study was a health facility-based study. Also, most of the private health facilities were not consistent blood donation centers and therefore were not considered in the study as their inclusion could have constituted a bias. Our findings will still provide the basis for the implementation of donor safety strategies in Minna.

## Conclusion

In conclusion, we observed a high seroprevalence of 96.2% of CMV in Minna among blood donors with a significant proportion (2.6%) capable of transmitting CMV infection to blood recipients. But since up to 96.2% of blood donors are seropositive for CMV, it would seem superfluous to screen blood donors for CMV for all transfusions, as few seronegative blood units would be available for transfusion. The majority of the blood donors were anaemic. Prospective blood donors for immunocompromised patients, however, should be screened for CMV. The quality of screening for anemia should be improved.

### What is known about this topic

- Transfusion is a lifesaving therapeutic intervention - However, many preventable errors may make this a hazardous procedure;
- Cytomegalovirus is transmissible through blood transfusion, among other parenteral routes, however, donor screening for CMV is not routinely undertaken in Nigeria;
- CMV infection is widely spread among the human population but not commonly known as most CMV infections are asymptomatic and therefore commonly go undiagnosed.

### What this study adds

- Combined CMV IgG and CMV IgM antibodies are detected in 2.6% of blood donors;
- Blood donors with monthly income level < ₦18000 are less likely to be CMV-positive than those with higher income;
- More than half of the study participants (blood donors) (65.8%) were anaemic i.e. had Hb below the normal range (i.e. Hb < 12g/dl) and majority were family replacement blood donors.

## Competing interests

The authors declare no competing interests.
